# Klotho and PPAR Gamma Activation Mediate the Renoprotective Effect of Losartan in the 5/6 Nephrectomy Model

**DOI:** 10.3389/fphys.2018.01033

**Published:** 2018-08-02

**Authors:** Edgar Maquigussa, Josne C. Paterno, Gabriel H. de Oliveira Pokorny, Mariana da Silva Perez, Vanessa A. Varela, Antônio da Silva Novaes, Nestor Schor, Mirian A. Boim

**Affiliations:** Nephrology Division, Department of Medicine, Federal University of São Paulo, São Paulo, Brazil

**Keywords:** klotho, angiotensin II, PPAR-γ, chronic kidney disease, hypertension

## Abstract

Renin angiotensin system (RAS) blockade reduces the progression of chronic kidney disease (CKD) independently of its antihypertensive effect. Ang II-induced fibrosis can be mediated by molecules such as klotho, peroxisome proliferator-activate receptor γ (PPAR-γ), and the Wnt/β-catenin pathway; however, the interaction among these molecules and RAS activation is not completely known. The aim of this study was to investigate a possible link between RAS, PPAR-γ, and Klotho in the 5/6 nephrectomy (NX) animals. NX rats presented hypertension that was blunted by both losartan and propranolol, however, only losartan was able to reduce the expression levels of fibronectin FSP1 and TGF-β in the remnant kidney. The anti-fibrotic Klotho and PPAR-γ were reduced in the remnant kidney, and losartan, but not propranolol, restored their levels. In contrast, the profibrotic Wnt 7a and Wnt 3 were upregulated and losartan prevented the increase in Wnts. *In vitro*, Ang II induced a decrease in both klotho and in PPAR-γ in Madin-Darby canine kidney (MDCK) cells, and this effect was blunted by losartan. However, klotho expression was increased by pioglitazone, an agonist of PPAR-γ, and suppressed by BADGE, an antagonist of PPAR-γ, suggesting that the effect of Ang II downregulating klotho is mediated by PPAR-γ. These data suggest that activation of the Wnt pathway together with downregulation of PPAR-γ that in turn suppresses klotho contribute to potentiating the profibrotic effect of Ang II.

## Introduction

Renal fibrosis is recognized as the final common pathway leading to chronic kidney disease (CKD) (Ruggenenti et al., 2012). The development of renal fibrosis results from an imbalance between profibrotic and antifibrotic pathways. Fibroblast activation and the epithelial mesenchymal transition (EMT) play an important role in extracellular matrix (ECM) deposition contributing to fibrosis. These profibrotic processes are modulated by the upregulation of genes related to fibrosis, including Angiotensin II (Ang II), TGF-β, Wnt, connective tissue growth factor (CTGF), andα-smooth muscle actin (α-SMA), together with the downregulation of antifibrotic genes, such as klotho, peroxisome proliferator-activate receptor γ (PPAR-γ), and bone morphogenetic protein (BMPs) (Hu et al., 2013).

The Klotho gene encodes a single-pass transmembrane protein that plays an important role in aging processes, however, its effects go beyond the aging process. Renal klotho is a key molecule in calcium metabolism that involves FGF23 responses (Urakawa et al., 2006). Moreover, the soluble form of klotho has been implicated in many pathophysiological mechanisms of renal diseases (Koh et al., 2001). Klotho is markedly decreased in acute kidney injury and in CKD patients (Hu et al., 2013). Drew et al. (2017) demonstrated an association between low levels of soluble klotho and the rate of decline of kidney function. Thus, soluble klotho acts as an endocrine factor with diverse functions including endothelial protection, reducing inflammation and fibrosis, and evidence has suggested that this protection is mediated by a suppression of the pro-fibrotic Wnt signaling pathway by klotho (Zhou et al., 2013). The Wnt protein family binds to *Frizzled* receptors and transmits their signals to induce the transcription of pro-fibrotic genes (Tan et al., 2014), and thus, klotho may act as an endogenous modulator of the Wnt pathway.

On the other hand, klotho expression can be modified by other molecules, including the PPAR-γ and Ang II (Mitani et al., 2002; Zhou et al., 2010; Yoon et al., 2011; Lin W. et al., 2017 ; Raeisi et al., 2017). Classically, PPAR-γ is associated with regulation of lipid and glucose metabolism, but PPAR-γ also has non-metabolic effects, including inhibition of fibrosis and cell cycle regulation (Ma et al., 2001). It is not clear, however, whether these PPAR-γ effects are mediated by klotho. In contrast to PPAR-γ, Ang II downregulates klotho expression (Yoon et al., 2011); nevertheless, a possible mechanism of Ang II inducing fibrosis involving klotho remains incompletely understood. Therefore, we examined the hypothesis that Ang II can induce fibrosis by suppressing the Klotho/PPAR-γ pathway in a CKD rat model and the crosstalk among the angiotensin II, klotho, and PPAR-γ in an *in vitro* model.

## Materials and Methods

The experimental protocol was approved by the Ethical Committee of the Federal University of São Paulo (CEP 726687, UNIFESP, São Paulo, Brazil). The study used 12-week-old male Wistar rats (150–200 g) supplied by the animal facility of the Universidade Federal de São Paulo. The rats were housed in cages with *ad libitum* access to standard rat chow and tap water, in a temperature-controlled environment (23°C) with a 12 h light/dark cycle.

### *In Vivo* Study

For 5/6 renal mass ablation (NX), rats were anesthetized with 40 mg/kg ketamine (Syntec, Hortolândia, Brazil) and 20 mg/kg xylazine (Syntec, Hortolândia, Brazil), i.p. The right kidney was surgically removed, and two or three branches of the left renal artery were individually ligated resulting in the infarction of two-thirds of the left kidney. Sham-operated rats were subjected to anesthesia and manipulation of the renal pedicles. The animals were separated into four groups: Sham (CTL; *n* = 5), 5/6 nephrectomy (NX; *n* = 5), NX treated with losartan (LOS; 20 mg/kg/day in the drinking water, *n* = 5), and NX treated with propranolol (PROP, 20 mg/kg/day in the drinking water, *n* = 5). The dosage of losartan and propranolol were selected based on previous studies (Lebel et al., 2006; Kobuchi et al., 2014). In the present study, two doses were tested (50 and 20 mg/kg/day) in order to verify the best one to decrease blood pressure. Both doses were effective to reduce blood pressure, then the lower dose of 20 mg/kg/day was chosen. The losartan and propranolol were purchased from Sigma-Aldrich and Magister Medicamentos, respectively. Both treatments were started 1 day after nephrectomy. All animals were sacrificed 8 weeks after the onset of treatments.

Periodically, the systolic blood pressure was measured by tail-cuff plethysmography (ADInstruments, Sydney, NSW, Australia). One day before euthanization, the animals were placed in metabolic cages (Tecniplast, Buguggiate, Italy) for 24 h urine collection. At completion of the experimental protocol, the animals were anesthetized with ketamine and xylazine, and blood samples were collected from the abdominal aorta and the remnant kidney was excised. Animals were euthanized via anesthetic overdose (160 mg/kg ketamine and 80 mg/kg xylazine; Syntec, Hortolândia, Brazil). Urine protein excretion (Sensiprot, Labtest, Lagoa Santa, Brazil) and blood creatinine (Creatinine CE, Labtest) and urea nitrogen (BUN; Urea CE, Labtest) were measured using a colorimetric assay. For the mRNA and protein expression analyses, the kidney samples were immediately frozen in liquid nitrogen and kept at -80°C until use. For histochemical and immunohistochemical analyses, the kidney samples were fixed in tamponed formaldehyde (Merck KGaA, Darmstadt, Germany) and, following several washes in ethanol (Merck KGaA, Darmstadt, Germany) and xylene (Labsynth, Diadema, Brazil), the samples were embedded in paraffin wax (Labsynth, Diadema, Brazil).

### Light Microscopy Studies

Histological analyses with hematoxylin and eosin and picrosirius red were performed, as previously described (Maquigussa et al., 2015). The fibrotic area stained with picrosirius solution was quantified using Leica application suite (LAS V3.8, Leica Microsystems, Wetzlar, Germany). Five images per animal were analyzed.

### Immunohistochemistry

The kidney slices were deparaffinized and rehydrated. To expose the antigens, the kidney sections were boiled in a target retrieval solution [citrate buffer (pH 6.0)] for 30 min. Endogenous peroxidase activity was blocked with 3% H_2_O_2_ (Labsynth, Diadema, Brazil) for 10 min at room temperature. Non-specific binding was prevented by incubating the sections with a protein blocker (Dako, Carpinteria, CA, United States). The sections were incubated overnight at 4°C with klotho primary antibodies (Anti-Klotho, Abcam, Cambridge, MA, United States). Following washing with Tris-buffered saline (TBS) [50 mM Tris, 150 mM NaCl], the sections were incubated with a horseradish peroxidase (HRP)-conjugated polymer (Dako, Carpinteria, CA, United States) for 30 min at room temperature. The slides were rinsed with TBS, and the sites of antibody–antigen binding were visualized with 3,3′-diaminobenzidine (Dako, Carpinteria, CA, United States). The sections were lightly counterstained with hematoxylin. The analyses were performed using light microscopy (Eclipse 2000 camera Nikon DS-Fi2). Quantification of klotho immunostaining was performed by analyzing five images of the renal cortex for each animal and the intensity of staining was quantified using the optical density (OD) function of the software (LAS V3.8, Leica microsystems, Wetzlar, Germany) and expressed as percentage of positive pixels in the analyzed area.

### Western Blot Analysis

The kidney fragments were homogenized using a Precellys 24 homogenizer in ice-cold buffer [50 mM TRIS (Sigma-Aldrich, St. Louis, MO, United States), 150 mM NaCl (Labsynth, Diadema, Brazil)], 1.0% nonidet-P-40 (Bio-Rad Laboratories, Hercules, CA, United States), 0.5% sodium deoxycholate (Sigma-Aldrich, St. Louis, MO, United States), 0.1% SDS, (pH 8.0; Sigma-Aldrich, St. Louis, MO, United States) containing protease inhibitors (AEBSF, aprotinin, bestatin, E-64, leupeptin, pepstatin A; Protease Inhibitor Cocktail; Sigma-Aldrich, St. Louis, MO, United States). Total protein was quantified using a modified Lowry method (Bio-Rad DC protein assay reagent; Bio-Rad Laboratories). Protein samples (50 μg) were separated according to size by 12% SDS-PAGE and electroblotted onto nitrocellulose membranes (GE Life Sciences, Little Chalfont, United Kingdom). The membrane blots were probed with primary antibodies overnight at 4°C and with HRP-conjugated secondary antibodies for 1 h at 4°C. The primary antibodies were obtained from the following sources: mouse monoclonal anti-β actin (Sigma-Aldrich, St. Louis, MO, United States) and mouse monoclonal Anti-Klotho (Anti-Klotho, Abcam, Cambridge, MA, United States). Next, the membranes were incubated with goat anti-rabbit (GE Life Sciences) and rabbit anti-mouse (Sigma-Aldrich, St. Louis, MO, United States) HRP-conjugated secondary antibodies. The protein bands were visualized using the Immobilon Western HRP substrate (Millipore). The obtained bands were quantified using Uvitec analyses software (Uvitec Limited, Cambridge, United Kingdom).

### *In Vitro* Study

Madin-Darby canine kidney (MDCK), a cell line that display mostly distal tubular features, was obtained from the American Type Culture Collection (ATCC, Manassas, VA, United States). The MDCK cells were cultured in Dulbecco’s modified Eagle’s medium (DMEM, Gibco, Grand Island, NE, United States) supplemented with 5% fetal bovine serum (Gibco, Grand Island, NE, United States). At semi-confluence, the cells were incubated with the respective drug, angiotensin II (10^-12^, 10^-10^, 10^-8^, and 10^-6^ mol/L), losartan (10^-8^ mol/L), pioglitazone, an agonist of PPAR-γ (10^-7^, 10^-6^, and 10^-5^ mol/L), and/or BADGE, an antagonist of PPAR-γ (10^-3^ mol/L), for 24 h. All of the reagents were obtained from Sigma-Aldrich. Six samples were included per experimental condition in the studies.

### Quantitative Polymerase Chain Reaction (qPCR)

Total RNA was isolated using the phenol and guanidine isothiocyanate-cesium chloride method with TRIzol (Ambion, Carlsbad, CA, United States), according to the manufacturer’s instructions. Total RNA (2 μg) was treated with DNase (RQ1 RNase-free DNase; Promega, Madison, WI, United States) to avoid genomic DNA contamination and reverse-transcribed into cDNA by adding a mixture containing 0.5 mg/ml of oligo(dT) (Invitrogen Life Technologies, Carlsbad, CA, United States), 10 mM DL-dithiothreitol (Invitrogen Life Technologies, Carlsbad, CA, United States), 0.5 mM deoxynucleoside triphosphates (Invitrogen Life Technologies, Carlsbad, CA, United States) and 200 units of reverse transcriptase enzyme (SuperScript RT II; Invitrogen Life Technologies, Carlsbad, CA, United States). The mRNA expression levels were estimated using qPCR (QuantStudio 7; Applied Biosystems, Foster City, CA, United States) by TaqMan or SYBR Green qPCR methods. Specific TaqMan Assay primer sets (Applied Biosystems, Foster City, CA, United States) were purchased for the following genes: Wnt3-rat (Rn01471586_m1); Wnt7a-rat (Rn01425352_m1); GSK3β-rat (Rn00583429_m1); PPARγ-rat (Rn00440945_m1), klotho-rat (Rn00580123_m1), βactin-rat (Rn00667869_m1), PPARγ-canine (Cf02625544_m1), klotho-canine (Cf03023880_g1), and βactin-canine (Cf02644567_m1). The following forward and reverse primers, respectively, were used: FSP1 (ATACTCAGGCAACGAGGGTGACAA and GTCCCTGTTGCTGTCCAAGTTGTT); fibronectin (AATGAGAGTGATAACGCTGATGTCA and TCCCCATTTTTGAAGATTTTGTG); TGF-β (TGACGTCACTGGAGTTGTACGG and AACTATTGCTTCAGCTCCACAGAGA); and βactin (CCTCTATGCCAACACAGTGC and ACATCTGCTGGAAGGTGGAC). All primers were synthesized by Integrated DNA Technologies (Coralville, IA, United States). The comparative CT method (ΔΔCT) was employed to quantify gene expression, and the relative mRNA quantification was calculated as 2^-ΔΔCT^. The mRNA expression levels were normalized to β-actin expression, which was used as an endogenous control.

### PPARγ Activation

The MDCK cells were incubated with Ang II (10^-10^ mol/L); Ang II and losartan (10^-6^ mol/L); or Ang II and BADGE (10^-3^ mol/L) for 24 h. Nuclear extracts from the cell culture were obtained using a nuclear extraction kit (Sigma-Aldrich, St. Louis, MO, United States). To quantify PPARγ activation, nuclear extract was measured using the Trans-AM PPARγ kit according to the manufacturer’s instructions (Active Motif, Carlsbad, CA, United States).

### Statistical Analysis

The results are expressed as the mean ± standard error. The data were analyzed by SigmaStat 2.0 software (Systat Software Inc., San Jose, CA, United States) using one-way analysis of variance (ANOVA) followed by Tukey’s test. *P* < 0.05 was considered to indicate a statistically significant difference.

## Results

### Blood Pressure and Renal Function

**FIGURE 1:**
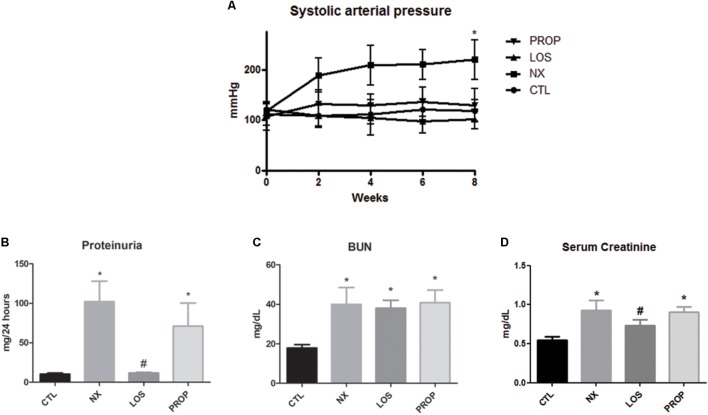
Blood pressure and renal function. Evaluation of systolic arterial pressure **(A)**, proteinuria **(B)**, blood urea nitrogen **(C),** and serum creatinine **(D)**. The data are expressed as the mean ± standard error of the mean. Groups: CTL (*n* = 5), NX (*n* = 5), LOS (*n* = 5), and PROP (*n* = 5). ^∗^*P* < 0.05 vs. CTL, ^#^*P* < 0.05 vs. NX.

As expected, the animals subjected to the 5/6 NX presented hypertension within 2 weeks after ablation (**Figure 1A**) and blood pressure remained higher than the sham group (107 mmHg) during the next 6 weeks (*P* < 0.05). The development of hypertension was prevented in both the losartan-treated group and propranolol-treated group. Within 8 weeks of 5/6 ablation, rats developed renal failure characterized by increased proteinuria (**Figure 1B**), elevation in serum BUN (**Figure 1C**), and serum creatinine (**Figure 1D**). The proteinuria and increased serum creatinine levels were blunted by losartan treatment (*P* < 0.05). In contrast, propranolol did not significantly decrease these parameters. BUN remained elevated in all NX groups.

### EMT and Renal Fibrosis

**FIGURE 2:**
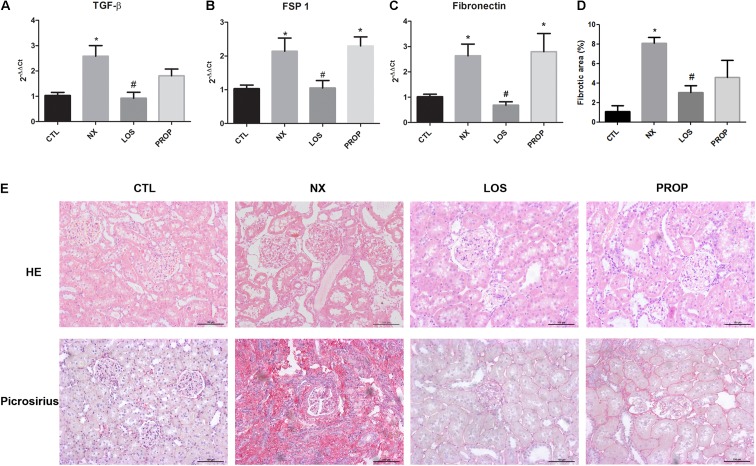
Kidney histology and EMT markers. Expression of TGF-β and EMT marker genes by qPCR **(A–C)**. Histological analysis of kidney sections stained with HE. Histological analysis of kidney sections stained with picrosirius red, showing collagen deposition. Magnification, ×40. Quantitative data of interstitial fibrosis and graphical representation of histological analysis shown in **D,E**. The data are expressed as the mean ± standard error of the mean. Groups: CTL (*n* = 5), NX (*n* = 5), LOS (*n* = 5), and PROP (*n* = 5). ^∗^*P* < 0.05 vs. CTL, ^#^*P* < 0.05 vs. NX.

The increase in the levels of EMT inducer (TGF-β) and markers (FSP1 and fibronectin) observed in NX animals was totally prevented by losartan but not by propranolol treatment (**Figures 2A–C**). These data corroborate those from the literature in that renin angiotensin system (RAS) activation can stimulate fibrosis mediators independently of inducing blood hypertension. On the other hand, reducing blood pressure has beneficial effects on the kidneys and, in fact, kidney histology of NX animals showing glomerular and tubular hypertrophy was significantly improved by LOS and also by PROP (**Figure 2E**). The collagen deposition was lower in the LOS and PROP groups compared with NX animals as indicated by the Picrosirius red staining (**Figures 2D,E**).

### Klotho and PPARγ

**FIGURE 3:**
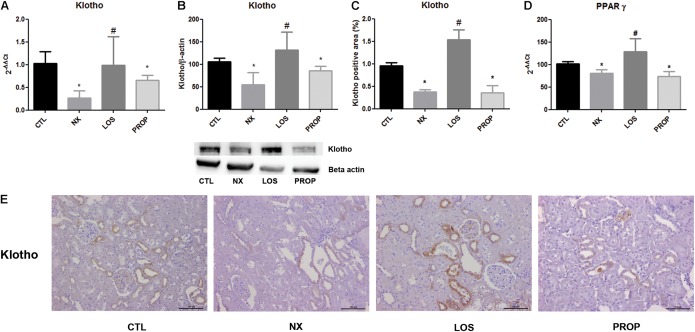
Gene expression of klotho **(A)** and PPAR-γ **(D)**. Western blot analysis of klotho protein expression **(B)**. Immunohistochemistry staining for klotho and representative microphotographs (magnification, ×40) showing cortical sections of the kidney and the quantitative analyses **(C,E)**. The data are expressed as the mean ± standard error of the mean. Groups: CTL (*n* = 5), NX (*n* = 5), LOS (*n* = 5), and PROP (*n* = 5). ^∗^*P* < 0.05 vs. CTL, ^#^*P* < 0.05 vs. NX.

Klotho mRNA and protein expression were both reduced in NX animals (**Figures 3A,B**). Klotho suppression was prevented by LOS but not by PROP. The immunohistochemistry showed the presence of klotho mainly in the renal tubules in CTL animals, and the expression was decreased in NX animals (**Figure 3E**). The suppression of klotho in the NX group was reversed by losartan treatment (**Figure 3C**). PPARγ mRNA was decreased in the NX group, and only LOS was able to reverse this effect (**Figure 3D**).

**FIGURE 4:**
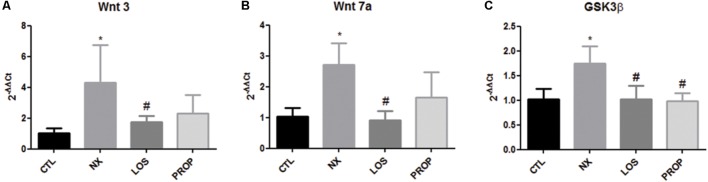
Gene expression. Gene expression of Wnt 3 **(A)**, Wnt 7a **(B)**, GSK3β **(C)**. The data are expressed as the mean ± standard error of the mean. Groups: CTL (*n* = 5), NX (*n* = 5), LOS (*n* = 5), and PROP (*n* = 5). ^∗^*P* < 0.05 vs. CTL, ^#^*P* < 0.05 vs. NX.

Soluble klotho acts as endocrine factor through mechanisms involving Wnt signaling. We examined the gene expression of two Wnt ligands and of the downstream GSK3β regulatory effect enzymes. As shown in **Figure 4**, both Wnt 3 and Wnt 7a were increased in the NX group. LOS prevented Wnt 3 and Wnt 7a elevation. GSK3β mRNA was increased in the NX group, which was blunted by both LOS and PROP treatments (**Figure 4C**).

### Effect of Angiotensin II on Klotho Expression in MDCK Cells

**FIGURE 5:**
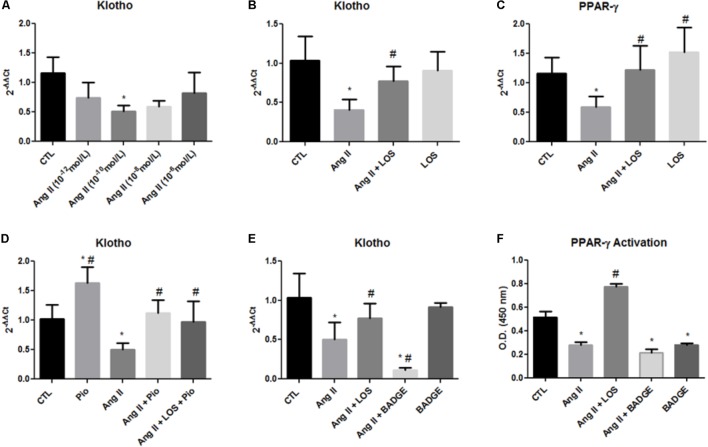
Gene expression and PPAR-γ activation. Gene expression of klotho and PPAR-γ in MDCK cells **(A–E)**. PPAR-γ activity as assessed by an ELISA of MDCK cells **(F)**. The data are expressed as the mean ± standard error of the mean. ^∗^*P* < 0.05 vs. CTL, ^#^*P* < 0.05 vs. Ang II. (*n* = 6).

To mimic the overactivation of intrarenal RAS in the remnant kidney, an *in vitro* model using MDCK cells was utilized and the effect of Ang II added to the culture medium on klotho expression was analyzed. A dose–response curve (**Figure 5A**) showed that cells incubated with different doses of Ang II for 24 h presented decreased klotho expression with significance at a dose of 10^-10^ mol/L. Losartan reversed the klotho suppression induced by Ang II (**Figure 5B**).

It has been shown that both klotho and PPAR-γ are predominantly expressed in distal tubules (Lin W. et al., 2017), and thus, we also evaluated the effect of Ang II in PPAR-γ expression in MDCK cells (from distal tubules). Similar to klotho expression, Ang II also decreased PPAR-γ expression and LOS inhibited this effect (**Figure 5C**).

To confirm that PPAR-γ can regulate klotho expression, we used an agonist of PPAR-γ, i.e., pioglitazone. The cells were stimulated with pioglitazone (10^-5^ mol/L) for 24 h. Pioglitazone increased klotho in unstimulated control cells and reversed klotho suppression caused by Ang II; moreover, losartan did not potentiate this effect (**Figure 5D**). The MDCK cells were also treated with Ang II and BADGE, an antagonist of PPAR-γ, and we assessed klotho expression by qPCR. As shown in **Figure 5E**, BADGE treatment potentiated the Ang II effect on klotho expression and the treatment with BADGE alone did not significantly alter these parameter. Finally, PPAR-γ binding activity was evaluated in nuclear extracts of MDCK with DNA-binding assays. The cells incubated with BADGE presented decreased PPAR-γ activation, these result indicate the effect of BADGE in blocks the activity of PPAR-γ (**Figure 5F**). Moreover, Ang II decreased PPAR-γ activation, and LOS reversed this effect (**Figure 5F**).

## Discussion

The results presented in this study support the hypothesis that Ang II can suppress klotho expression through PPAR-γ downregulation. Our results demonstrated that the expression of klotho was reduced in the remnant kidney of the 5/6 renal mass ablation model of CKD in agreement with other (Takenaka et al., 2015; Zhou et al., 2015b; Muñoz-Castañeda et al., 2017). The decrease in klotho expression could be associated with loss of renal mass, however, the mechanisms of klotho suppression can be more complex, since many events associated with CKD including uremic toxin, oxidative stress, proinflammatory cytokines and RAS activation are able to downregulate klotho (Hu et al., 2012, 2013). However, in the present study, the suppression of renal klotho in NX animals was fully reversed by losartan. This result suggests that Ang II has the potential ability to downregulate klotho and that this effect was independent of the hypertensive effect of Ang II, since the antihypertensive drug propranolol induced no change in klotho expression. Similar results were observed *in vitro* using MDCK cells. The U-shaped format of the dose–response curve for the klotho expression effect of Ang II suggests that at higher concentrations of Ang II can promote non-specific effects such as stimulation both AT1 and AT2 receptor. Exogenous addition of Ang II (10^-10^ mol/L) caused a reduction in klotho expression, and this effect was blunted by losartan. These results add evidence for a relationship between Ang II and klotho.

It is well described that losartan is renoprotective and reduces the progression of renal fibrosis induced by Ang II. In agreement with previous findings (Raeisi et al., 2017), our results point out that the beneficial effect of losartan can be attributed to an upregulation of klotho expression. Klotho is capable of reducing fibrosis by inhibiting fibroblast growth factor-2 (FGF2) signaling (Guan et al., 2014) and by inhibiting the Wnt pathway (Satoh et al., 2012). The process of renal fibrogenesis mediated by the Wnt/β-catenin pathway results in activation of profibrotic genes such as PAI-1 and FSP1 (He et al., 2009; Lin X. et al., 2017). Here, we found that in parallel with klotho suppression, Wnt 7a and Wnt 3 expression was increased in the remnant kidney and losartan also blunted this effect, suggesting that the profibrotic effect of Ang II is, at least in part, mediated by activation of the Wnt pathway. The activation of Wnt signaling inhibits the activity of glycogen synthase kinase-3 (GSK3β) which induces the accumulation of dephosphorylated β-catenin in the cytosol and its translocation into the nucleus (Clevers and Nusse, 2012). Our results demonstrated that the expression of GSK3β was increased in the remnant kidney and it was blunted by both losartan and propranolol treatments. Phosphorylated levels of GSK3β protein were not verified in the present study, however, this unexpected result suggests the GSK3β can be regulated by alternative pathways other than by Wnt.

On the other hand, previous studies demonstrated that activation of the Wnt/β-catenin pathway increased gene transcription of the RAS components in the kidney (He et al., 2009; Zhou et al., 2015a), and thus, together with the results obtained in the present study, the interaction between the Wnt/β-catenin pathway and Ang II can occur in both directions.

In contrast, klotho is a negative regulator of the Wnt pathway because klotho is able to bind multiple Wnt ligands and inhibits Wnt-mediated gene transcription (Satoh et al., 2012; Zhou et al., 2013; Tang et al., 2016). These data suggest that the antifibrotic effects of losartan are mediated by a reduction of Wnt 7a and Wnt 3 together with a recovery of the low levels of klotho induced by Ang II.

Peroxisome proliferator-activate receptor γ exerts multiple metabolic and non-metabolic effects, including negative regulation of fibrosis inducers (Yang et al., 2009). Our results showed that renal PPAR-γ was decreased in the NX group indicating a deficiency of an antifibrotic factor. PPAR-γ suppression was prevented by losartan, and this effect was independent of the blood pressure normalization since propranolol treatment did not change PPAR-γ expression in spite of its antihypertensive effect. This result is in agreement with previous findings that Ang II can downregulate PPAR-γ (Min et al., 2010). Besides the suppression of PPAR-γ gene transcription, we demonstrated, with an *in vitro* model, that Ang II decreased PPAR-γ activation in MDCK cells. Again, these effects were inhibited by losartan. The AT1 receptor blockade is associated with improvement of blood pressure and fibrogenesis outcomes, and the present results suggest that these effects can be associated at least in part through PPAR-γ activation (Bennett et al., 2004; Halabi et al., 2008). Therefore, it was demonstrated here that the beneficial renoprotective effects of losartan were mediated by a recovery of both klotho and PPAR-γ levels, two antifibrotic factors that are downregulated in models of CKD (Sung et al., 2004; Manya et al., 2010; Hu et al., 2011).

In addition, it has been suggested that renal klotho is regulated by PPAR-γ (Zhang et al., 2008), since PPAR-γ can bind to the non-canonical peroxisome proliferator response element (PPRE) to activate the klotho gene (Zhang et al., 2008). Moreover, Lin W. et al., 2017 demonstrated that PPAR-γ acetylation increases both membrane and secreted soluble forms of klotho. The present study showed that pioglitazone, a PPAR-γ agonist, induced klotho expression and reversed the effect of Ang II on klotho levels in MDCK cells. Similarly, the PPAR-γ antagonist BADGE potentiated the suppression of klotho induced by Ang II in MDCK cells. Taken together, these results suggest that blockadeof the Ang II receptor can restore klotho expression through PPAR-γ activation and revealed that PPAR-γ can be an additional link between Ang II and klotho.

## Conclusion

In conclusion, the data of the present study suggest that the renal antifibrotic effect of losartan can be mediated by at least two pathways: an upregulation of klotho via PPAR-γ and inhibition of Wnt/β-catenin signaling. The better knowledge on renal fibrosis mechanisms can offer therapeutic strategies those results in more promising outcomes in patients with CKD.

## Author Contributions

EM contributed to study design, data analyzing, discussion and preparation of the manuscript. JP, GP, MP, VV, and AN contributed to conducting the experiments. NS contributed to study design. EM and MB contributed to study design, discussion, reviewing and editing the manuscript.

## Conflict of Interest Statement

The authors declare that the research was conducted in the absence of any commercial or financial relationships that could be construed as a potential conflict of interest.
